# Detecting and Quantifying Forest Change: The Potential of Existing C- and X-Band Radar Datasets

**DOI:** 10.1371/journal.pone.0131079

**Published:** 2015-06-25

**Authors:** Mihai A. Tanase, Ismail Ismail, Kim Lowell, Oka Karyanto, Maurizio Santoro

**Affiliations:** 1 Cooperative Research Centre for Spatial Information, The University of Melbourne, Melbourne, Australia; 2 Gamma Remote Sensing AG, CH-3073 Gümligen, Switzerland; 3 Faculty of Forestry, Gadjah Mada University, Yogyakarta, Indonesia; Wuhan Botanical Garden,CAS, CHINA

## Abstract

This paper evaluates the opportunity provided by global interferometric radar datasets for monitoring deforestation, degradation and forest regrowth in tropical and semi-arid environments. The paper describes an easy to implement method for detecting forest spatial changes and estimating their magnitude. The datasets were acquired within space-borne high spatial resolutions radar missions at near-global scales thus being significant for monitoring systems developed under the United Framework Convention on Climate Change (UNFCCC). The approach presented in this paper was tested in two areas located in Indonesia and Australia. Forest change estimation was based on differences between a reference dataset acquired in February 2000 by the Shuttle Radar Topography Mission (SRTM) and TanDEM-X mission (TDM) datasets acquired in 2011 and 2013. The synergy between SRTM and TDM datasets allowed not only identifying changes in forest extent but also estimating their magnitude with respect to the reference through variations in forest height.

## Introduction

Forest structural characteristics estimation is a sensitive research topic since information on forest spatial extent, carbon stocks and their dynamics is needed for greenhouse gases flux estimation and, thus, policy development and implementation [1]. National based mechanisms aimed at reducing emissions from deforestation and forest degradation (REDD) rely on accurate estimation of such changes over time and space by measuring two key variables, i) the areal extent of deforestation and degradation and, ii) the carbon stock densities per unit area [2].

Monitoring such parameters at national level is a difficult task with remote sensing being considered a fundamental tool providing efficient solutions for developing and maintaining REDD and REDD+ programs [3–5]. Several methods have been used to measure historical deforestation and forest degradation some of them being based on remote sensing sources such as the global Landsat archive [6,7]. Such datasets proved sufficient to measure historical deforestation with adequate certainty for determining reference emissions [8]. However, monitoring carbon stock enhancements (e.g., afforestation or forest regrowth) beyond the point of canopy closure becomes less reliable using optical sensors due to signal saturation problems. Recently, it has been shown that radar backscatter-based methods allow for a much longer monitoring of forest regrowth (i.e., signal saturation occurs when forests reach 45–50 years) when compared to optical datasets (i.e., signal saturation occurs when forest reach 15–20 years) in semi-arid or boreal environments [9]. Furthermore, monitoring degradation using optical datasets is problematic since degraded areas are characterized by changes in forest structure rather than land cover type, i.e., forest canopy cover may not change significantly [10]. It is likely that initial degradation stages might be difficult to pick up using optical or radar backscatter-based remote sensing methods since the remaining trees may still provide sufficient canopy cover and respectively scattering elements to saturate the signal [11]. Quantifying the magnitude of positive forest change due to sustainable management, conservation or carbon stock enhancements would pose similar challenges when using optical or radar backscatter-based techniques.

An alternative approach is offered by synthetic aperture radar (SAR) interferometry (InSAR) which has become a major research topic because of its ability to monitor several bio- and geophysical parameters with high accuracy [12–21]. By measuring the relative phase of two SAR images acquired from two slightly different positions in space, radar interferometry provides information on the three dimensional distribution of a target. The sensitivity of the interferometric phase upon elevation is affected by radar frequency, image acquisition geometry, spatial separation between antennas and the temporal interval between image acquisitions [22]. However, the latter factor is negligible when the two images are acquired simultaneously (i.e., single-pass interferometry), thus preserving the interferometric phase from being affected by noise due to the decorrelation occurring between the satellite overpasses. Over vegetated terrain, the penetration of microwaves with short wavelengths (e.g., C- and X-band, 3–6 cm wavelength) is limited, thus implying that the backscattered signal originates above the terrain surface from within the canopy. The elevation of the scattering center depends on the forest density (amount and size of gaps) and the attenuation of the microwave through the canopy, i.e. drier and sparser canopies allow more penetration than dense and moist canopies, thus causing the scattering center to be located closer to the ground in the former case [23–25].

The first space borne single-pass interferometric acquisitions were provided by the Shuttle Radar Topography Mission (SRTM) in February 2000 at a nearly-global scale [26]. The C-band data acquired during the SRTM mission was used to produce a high-resolution digital elevation model (DEM) with a spatial resolution of 30 m. The assessment of the SRTM DEM showed that accuracy specifications were exceeded with the absolute elevation error being less than 6.5 m over bare areas [27,28] while the relative elevation error being less than 3.3 m [29]. However, over densely vegetated areas (i.e., forests) the elevation was up to 15 m higher when compared to the reference ground surface [28]. Such differences suggested that the SRTM elevation could be used as a proxy to derive estimates of vegetation height [30–33]. These studies exploited the difference between the SRTM elevation, which is influenced by vegetation, and an independent dataset of surface elevation. Since 2010 the TanDEM-X (TDM) complemented the TerraSAR-X mission to achieve a single-pass interferometer from space with the aim of generating a global digital elevation model with accuracy equaling or surpassing the high-resolution terrain information (HRTI-3) specification, i.e. 10 m absolute vertical accuracy, 10 m horizontal accuracy and 12 m spatial resolution [34].

The availability of two global datasets of terrain elevation with a temporal separation larger than 10 years provides an opportunity to estimate not only changes in land cover but also in forest structure (i.e., height) over the past decade. Indeed, recent studies showed that by combining SRTM and TDM datasets clear cuts can be identified in boreal environments [14]. Further studies have also shown that TDM derived heights can be used to accurately estimate biomass in boreal forests with root mean squared errors (RMSE) down to 16% [12,13]. However, such studies relied on information on ground elevation from lidar datasets which is a rare and expensive commodity over large areas such as those targeted by REDD and REDD+ projects. Recently, it was demonstrated that changes in carbon stocks in coniferous forests might be estimated relatively accurate by combining TDM and SRTM heights [35]. The aim of this study was to provide further evidence by analyzing the potential of SRTM and TDM datasets for detecting and quantifying forest changes in tropical and semi-arid environments using a slightly different approach. Specifically, changes in forest area over the last decade were identified and their magnitude was quantified based on differences in elevation measured with SAR interferometry which is ultimately related to changes in forest height. This could provide a more reliable indicator of forest degradation/growth when compared to methods relying on forest cover estimates.

## Study areas and ground measurements

**Fig 1:**
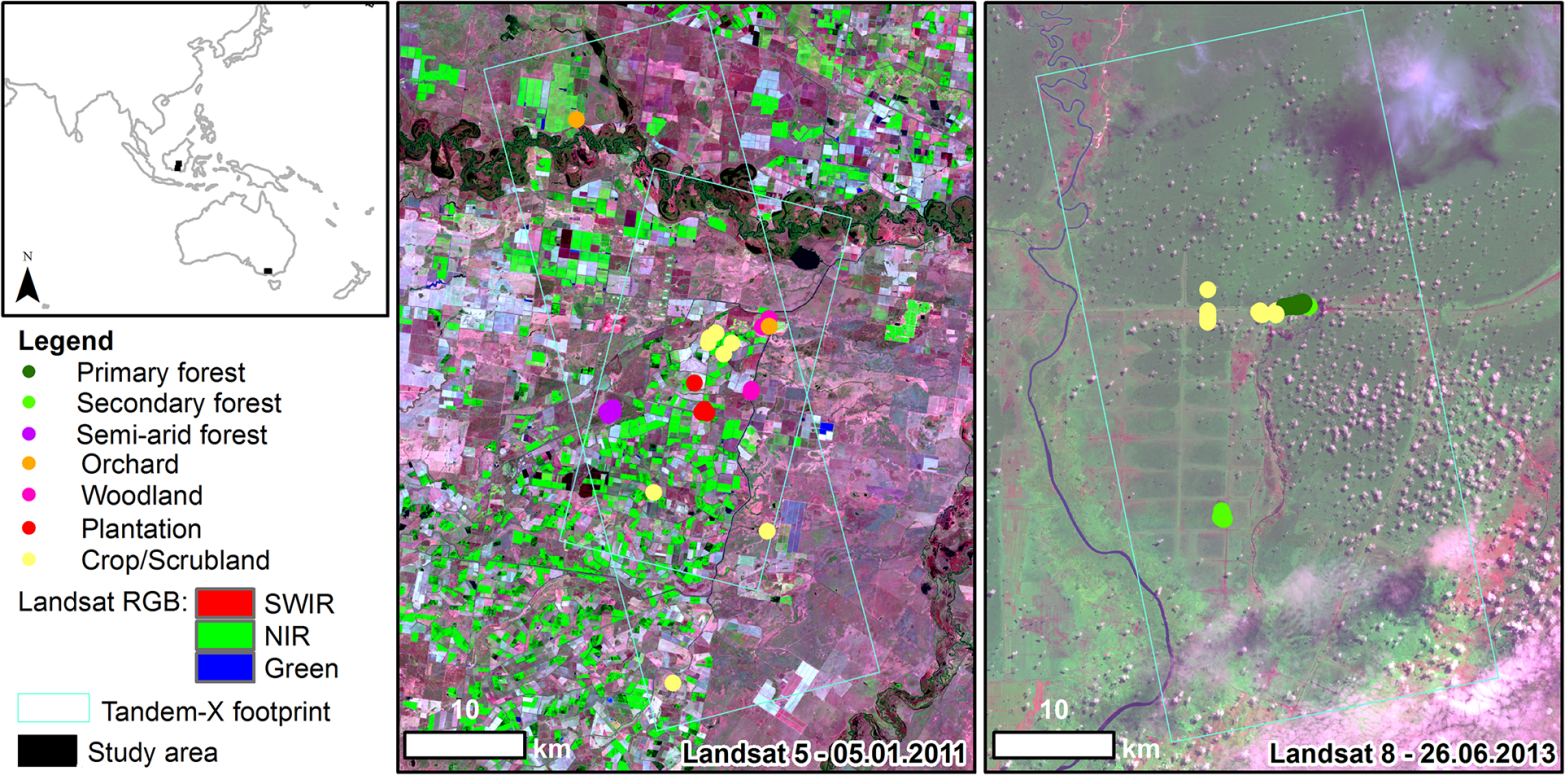
Overview of the study areas, the location of the ground sites and their vegetation type. Landsat images (center and respectively left panels) show the variability of land cover types inside TDM footprints showed in cyan. Due to scaling some ground sites appear as overlapped. Data available from the U.S. Geological Survey.

**Fig 2:**
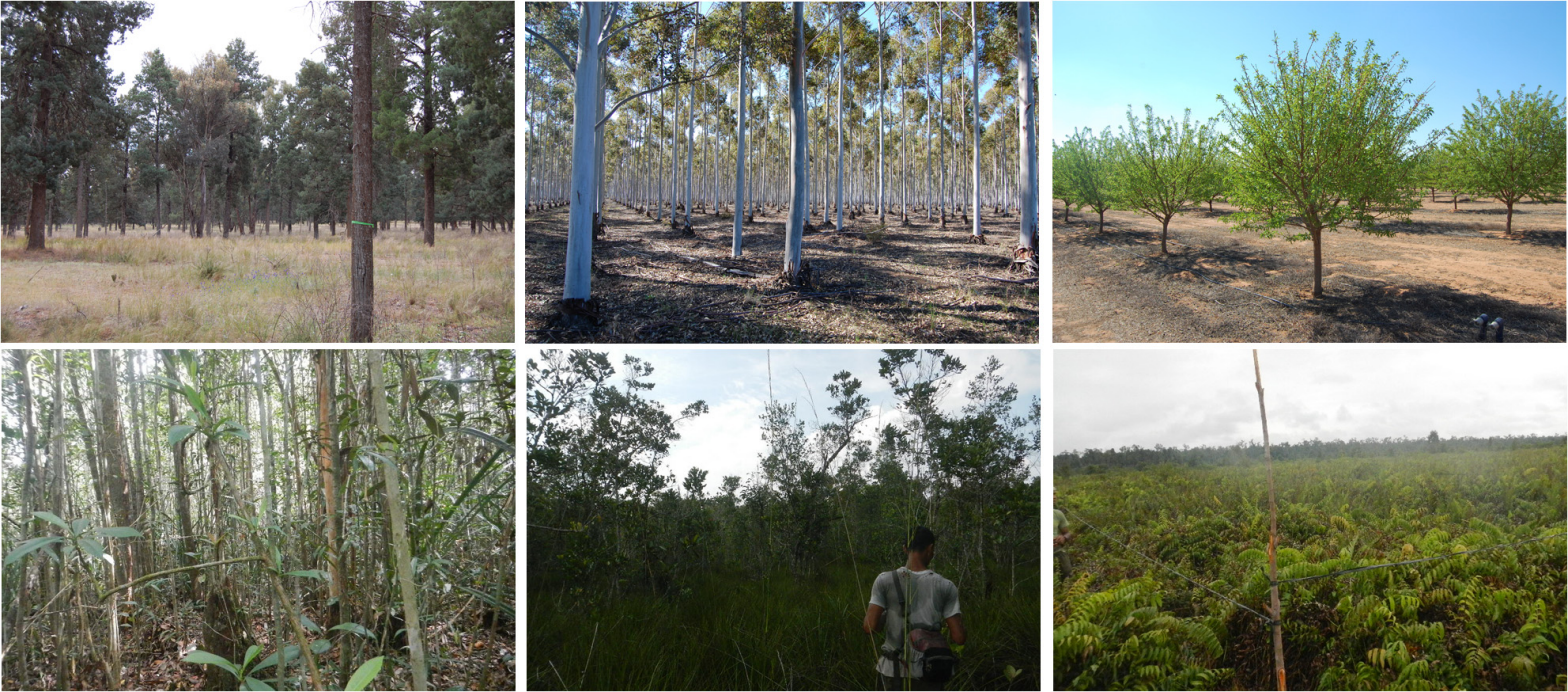
Vegetation types present in the study areas. Upper row presents typical vegetation types for the semi-arid study area (from left to right: white cypress forest, eucalyptus plantation and almond orchard). The lower row presents typical vegetation types for the tropical study area (from left to right: primary forest, secondary forest and scrubland).

Field and remote sensing data were available for two study areas corresponding to semi-arid and respectively tropical environments. The semi-arid study area was located in the western part of the Murrumbidgee catchment near the township of Darling Point, Australia (Fig 1). The area is characterized by agricultural and grazing farms interspersed with natural forests and forest plantations. The mean annual rainfall of 440 mm is distributed throughout the year. The topography is flat. The second study area was located in Kapuas, Central Kalimantan, Indonesia (Fig 1). The climate is tropical with a wet season from May to October and a dry season from November to April. The mean annual rainfall is about 2800 mm. The area was partly drained during mid-1990`s which resulted in a patchy land cover of forest, swamp, scrublands, and interspersed farmland. The forest is classified either as primary or secondary depending on the anthropic influence (Fig 2).

In the semi-arid study area seven forest sites dominated by white cypress pine (*Calitris glucophylla*) and four woodland sites dominated by grey box (*Eucalyptus microcarpa)* or black box trees *(Eucalyptus largiflorens)* were surveyed for tree height and diameter (S1 Table). In addition, six sites located in red river gum (*Eucalyptus camadulensis*) plantations, one olive orchard and one almond orchard as well as eight agricultural paddocks were surveyed for the mean tree height and respectively crop height (Fig 2). Tree height varied between 8 and 25 m while the height of orchard trees was around 4 m. Crop heights varied between 1 m for canola and 0.5 m for cereals. The field survey was conducted in September 2011. The surveyed area depended on the land cover type (Table 1). For the forest sites circular areas of 25 m radius were used while for woodlands the plot radius was reduced to 10 m since such a radius covered most of the trees present around the center of the plots. For the eucalyptus plantations and orchards squared areas (10 m × 10 m) were used since trees were highly homogeneous. Crop height for individual plants was measured at several locations within each paddock and averaged by paddock.

**Table 1:** Sampled vegetation types together with stand Lorey`s height for the two study areas.

Vegetation type	No. of sites	Sampled area (m^2^)	Lorey`s height (m)
Semi-arid study area			
White cypress pine forest	7	2000	10–16
Woodland	4	315	8–20
Plantation (Eucalyptus sp.)	6	100	10–21
Orchard	2	100	4–5 *
Crop	8	N/A	0.5–1*
Tropical study area			
Primary forest	20	100	8–17
Secondary forest	10	100	6–10
Scrubland	20	100	0–5*

* average height

In the tropical area 50 sites (10 m × 10 m) were field assessed for tree height and diameter in February 2014 (S2 Table). By land cover type, the sites were split between 20 primary forest sites, 10 secondary forest sites and 20 scrubland sites. For the primary forest, individual trees reached up to 27 m while for the secondary forest tree height was less than 10 m. Scrub areas were covered with low vegetation usually well below 5 m height. One should notice that due to access limitations primary forest sites were located along canals. This might explain the reduced number of big trees due to selective logging for household use.

Forest height was estimated through Lorey`s height (i.e., height weighed by basal area) which better represents stand height since it allows larger trees to contribute more to the mean. In the case of even age homogeneous stands (i.e., plantations, orchards) Lorey`s height largely equals mean stand height.

## InSAR data and processing methods

### TDM dataset

For each study area, the TDM data consisted of four images pairs. Over the semi-arid area, the image pairs were acquired concurrently with the field data sampling in September 2011. The pairs were acquired during both ascending and descending passes in dual polarization mode (VVHH and respectively VVVH). For the tropical study area, the image pairs were acquired during the ascending pass in single polarization mode (HH) between January and June 2013.

The across-track effective perpendicular baselines was between 58 m and 123 m. The height of ambiguity (HOA), which expresses the elevation difference corresponding to a full phase cycle and measures the phase sensitivity to elevation, varied between 58 and 145 m. One should notice that the largest HOA corresponds to shortest baseline and vice versa.

### DInSAR processing

All TDM datasets were processed using a Differential Interferometry approach (DInSAR), i.e. by using a DEM as a reference elevation to convert TDM phase difference to absolute height difference. Here, the SRTM DEM at 30 m spacing was used for both study areas. The aim of DInSAR processing was to identify and quantify the change component with respect to the SRTM DEM which contains information about the historic forest height for the year 2000. Over the Australian study area the SRTM DEM was enhanced at Geoscience Australia by removing artefacts and noise present in the original SRTM data [36]. A comparison carried out between the enhanced SRTM DEM and lidar-based DEMs available for small areas around the township of Darling Point showed that about 90% of the SRTM elevations are within ± 1m of the lidar elevation for cropping areas—i.e., likely representing the influence of crops on SRTM signal.

DInSAR processing included the removal of the phase component due to topography using a simulated phase based on the orbital information and the reference DEM. Thereafter, the DInSAR phase was unwrapped using the minimum cost flow algorithm, i.e., minimization of the total cost associated with phase discontinuities, with a weighting factor based on the local coherence [37]. Prior to phase unwrapping, the interferograms were filtered with an adaptive technique with a filtering function based on local fringe spectrum [38]. To avoid the strong phase noise which could affect the investigation, pixels with a coherence below 0.3 were masked out before unwrapping. The unwrapped phases were converted to elevation using the phase to height sensitivity. For clarity, we will refer to relative heights considering that the elevation in the SRTM dataset was subtracted from elevation in the TDM dataset. To keep low the phase noise in the interferograms, spatial averaging (referred to as multi-looking) was applied during the process of the interferogram generation; the InSAR phase was estimated based upon 30 pixels in the original TDM imagery, corresponding to multi-look factors of 6 and 5 in range and azimuth respectively. In this way, each pixel in the interferogram represented approximately 100 m^2^ areas, being comparable to the size of the smallest inventory units (Section 2). The relative heights were subsequently geocoded to Universal Transverse Mercator (UTM) coordinate system at 10 m pixel spacing. To assess the agreement between SRTM and TDM heights their difference was computed over stable cropping or scrubland areas. One should notice that in such areas TDM height should be largely equal to the SRTM height.

### Vegetation height estimation

For comparison with the field data, the relative heights were extracted and averaged over the ground sampled area. The relative heights were averaged over up to 20 pixels for the largest forest sites while two to three pixels were extracted for the smaller areas. For the small 10 m x 10 m sites the extraction was carried out over a larger 300 m^2^ area. The relative heights were extracted from all available TDM pairs and the highest value was paired with the ground plots. The rationale was that higher values corresponded to maximum difference in the scattering centers of the SRTM and TDM datasets i.e., least X-band penetration due to polarization (i.e. VV vs. VH) or variations in environmental conditions (i.e. dry vs. wet) between TDM pairs. Indeed, when dual-polarized datasets were available the highest value corresponded to the cross-polarized channel over forested areas. For the tropical study area the relative heights were compensated for the negative elevation difference observed over stable scrubland areas (see the Results section). The relative heights were related to stand Lorey`s heights to evaluate the agreement between ground and remote sensing estimates. The root mean squared error (RMSE) the coefficient of determination (R^2^) and the estimation mean difference to ground measured height (i.e., bias) were computed for all land cover types and by land cover type for each study area. One should notice that relative heights as estimated from InSAR do not directly represent Lorey`s heights which are based on the height of tree tops. In contrast, InSAR heights represent the height of a so-called phase scattering center, where one can assume the total scattering from the vegetation to be concentrated. Depending on the forest canopy density, the height of the scattering center varies from the surface level (completely transparent canopy) to nearly the top of the canopy (dense and opaque canopy). The degree of penetration of the microwaves in the vegetation canopy and thus the elevation of the phase scattering center depend on canopy density, amount and size of gaps in the canopy and between trees, and dielectric properties of the vegetation. As a consequence elevation estimated with InSAR is expected to be considerably lower particularly for open canopy forests.

### Forest change mapping

To appreciate the capability of the TDM/SRTM combination to map forest change, the relative heights were classified to form a map of changes with respect to the reference year. Depending on their magnitude the relative heights were grouped into five classes (Table 2). Negative height was considered a sign of deforestation or forest degradation while positive height was considered a sign of forest growth or afforestation. Differences of ± 1 m were labeled as unchanged areas. Positive differences up to 3 m were also associated to unchanged areas to account for the lower X-band penetration when compared to C-band. Such positive relative heights would reflect only the difference in scattering center heights and not actual changes on the ground. The threshold was based on the difference in the scattering centers of the two wavelengths found for boreal forests [28]. Areas were labeled as deforestation when a negative relative height of at least 7 m was observed. The threshold was based on previous findings for clear-cut area detection in boreal forests using similar datasets [14]. Relative heights between—7 and -1 m were labeled as degraded forests while, using the opposite reasoning positive heights above 3 and 7 m were considered forest growth and afforestation, respectively. For the tropical study area these thresholds were compensated to account for the negative elevation differences observed over stable scrubland areas which were most likely caused by an active subsidence process (see the Results section).

**Table 2:** Thresholds used to map forest change in semi-arid and tropical areas. For the tropical area the thresholds were compensated for subsidence.

Class/Study area	Semiarid	Tropical
Deforestation	< -7 m	< -6 m
Degradation	-1 to -7 m	-6 to -1 m
Stable	-1 to 3 m	-1 to 4 m
Forest growth	3 to 7 m	4 to 8 m
Afforestation	> 7 m	> 8 m

## Results

Phase unwrapping was successful, with only about 0.01–0.25% of the pixels being masked out (coherence < 0.3) depending on the interferometic pair. Such pixels were located mostly over water, thus not affecting the analysis of the relative heights over vegetated areas. Relative heights derived from TDM data were consistent when considering all available interferometric pairs with an average standard deviation around the mean of 0.5 m for the tropical area (HH polarization). For the semi-arid area the variation around the mean for the retrieved forest heights was higher (i.e. ± 1.1 m) when considering all polarizations and decreased slightly when considering just one polarization (± 0.9 m for VV polarization). The increased height variability between interferometric pairs was attributed to the different polarizations (HH, VV and VH) and acquisition passes (ascending/descending) used. One should notice that, the uncertainty in the height estimate, corresponding to the uncertainty in the InSAR phase varied between 1.4 m and 3.8 m (2 m on average) depending on the interferometric pair and polarization [39]. The agreement between SRTM and TDM elevation tested over stable cropping or scrubland areas showed differences below 1 m (RMSE 0.7 m) with almost no bias (i.e., 0.1 m) for the semi-arid area. For the tropical area the difference in elevation was higher (RMSE 2.3 m) with a negative bias (i.e.,- 0.8 m). The higher RMSE and the negative bias observed for the tropical study area was explained by an active subsidence process due to drainage which is well document for drained peat lands [40].

**Fig 3:**
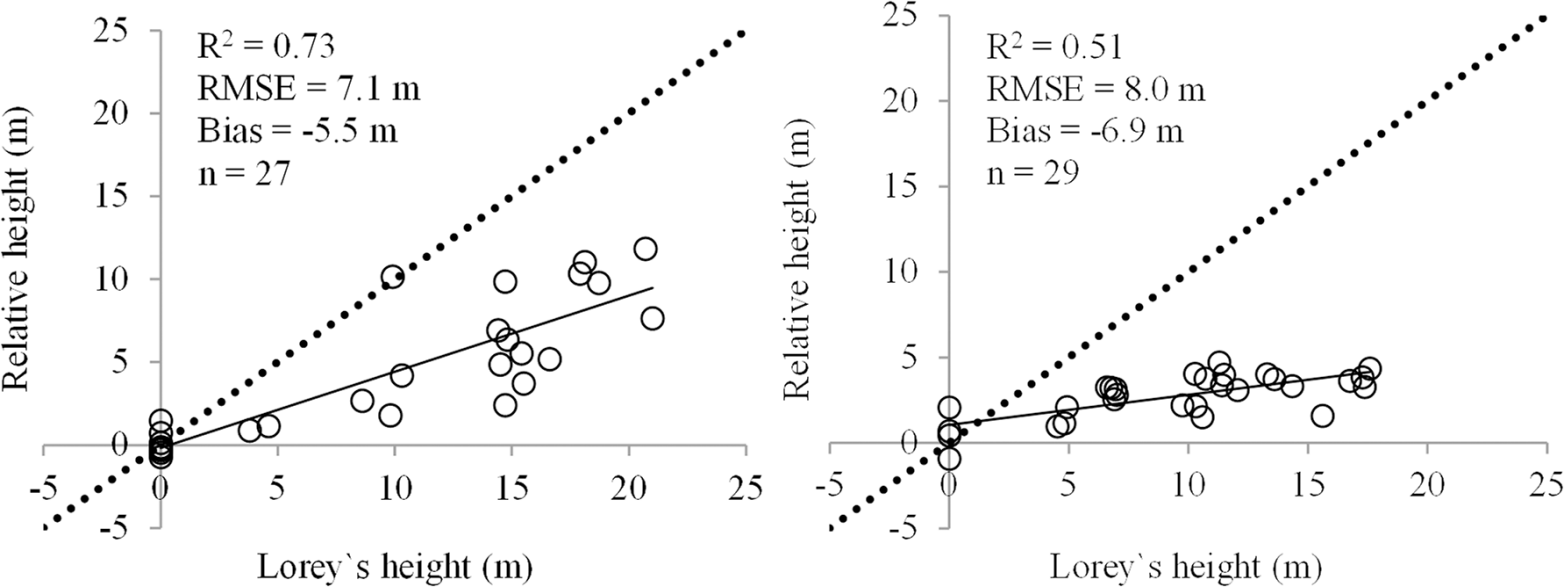
Overall statistic agreement between field and remotely sensed estimates of height for unchanged areas (i.e., sites affected by deforestation and forest degradation were excluded). Left panel represents the semi-arid study area whereas right panel represents the tropical study area.

The agreement between field measured heights and relative heights in the semi-arid study area was high when considering unchanged forests, new plantations and cropping areas, i.e. over 70% of relative heights`variability was explained by variations of the ground measured height (R^2^ = 0.73). For the tropical study area only a moderate agreement was observed (R^2^ = 0.51) when considering unchanged areas (i.e., forest and scrublands) as well as regrowth areas (Fig 3). The agreement increased (R^2^ = 0.60) when considering only forest and scrublands, i.e., areas with a stable land cover type between the overpass of SRTM and TDM missions. As expected, the InSAR height estimates were smaller with respect to the ground measurements (-5.5 to -6.9 m) since the phase scattering center is located below the top of the canopy for both C- and X-bands but somewhere above the ground (Fig 3). One should notice that for a perfect scenario, when X-band represents the top canopy height (low penetration) while C-band represents the ground surface (high penetration) the relative height (i.e. InSAR height) would be close the actual forest height. Otherwise, the InSAR height only represents the difference in penetration at the two wavelengths.

When analyzing just unchanged forest areas the difference between InSAR and ground height was about 10 m for the primary tropical forest and about 9 m for the semi-arid forest which represents about two thirds of the forest height (Tables 1 and 3). The low tree density, discontinuous canopy, absence of an understory layer and a dry environment allows for high penetration at both wavelengths which result in relatively large differences between ground and InSAR heights for semi-arid open forests. The higher differences observed for the tropical areas seems counterintuitive since one expects better results in such tropical environments were vegetation is dense and wet thus allowing for less penetration through the canopy. However, it seems that local conditions had a lesser than expected effect since the variation around the mean for the retrieved forest heights was low (i.e. ± 0.5 m) over the four TDM datasets analyzed in the tropical area. The lowest bias was observed for new forests/plantations with the difference between InSAR and ground height being around 6 m, about a third of the tree height (Tables 1 and 3). Such a higher agreement was the result of two factors: an accurate estimation of the topographic elevation from SRTM data (i.e., no vegetation was present at the time) and a decreased penetration of the TDM due to the continuous canopy and the increased vegetation water content i.e., such plantations are usually flood irrigated to boost productivity.

**Table 3:** Statistic agreement between field and remotely sensed estimates of height by land cover type for each study area. Sites affected by deforestation and forest degradation were excluded for the tropical study area.

Statistic	Semi-arid					Tropical		
	Forest	Woodland	Plantation	Orchard	Crops	Primary forest	Secondary forest	Scrubland
RMSE (m)	9.5	10.4	7.0	3.2	0.7	10.3	4.7	2.4
Bias (m)	-9.3	-9.9	-6.2	-3.2	0.1	-10.0	-4.4	-1.1
n	7	4	6	2	8	16	7	7

**Fig 4:**
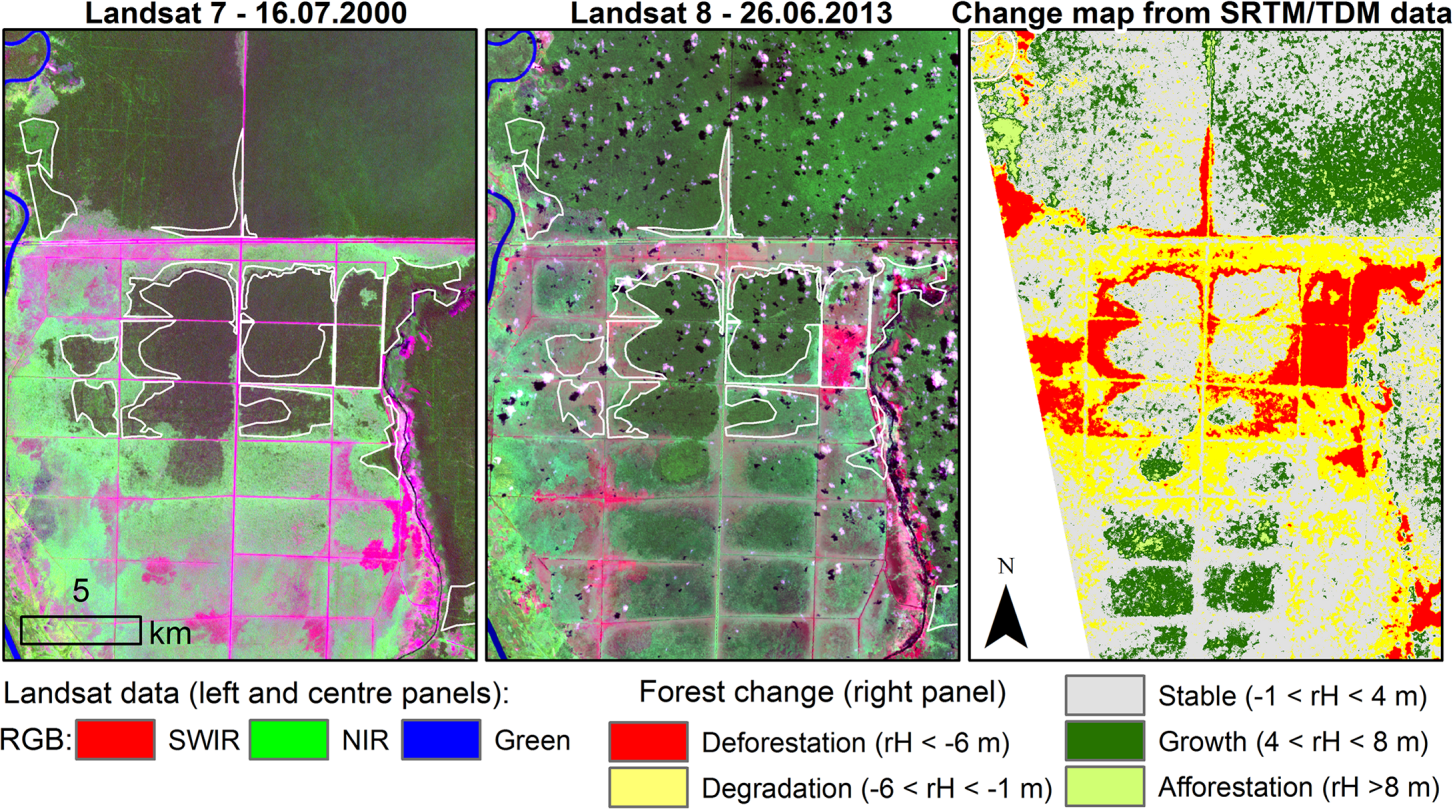
Subset of the tropical study area showing forest status (left and center panels) and forest change (right panel). Landsat data acquired in 2000 (left panel) and 2013 (center panel) show: forest areas in dark green, scrublands in light green and bare soil in magenta tones. Large areas affected by deforestation or afforestation (right panel) are highlighted in the Landsat images (left and center panels). Landsat data were available from the U.S. Geological Survey.

For the semi-arid study area the detected changes were limited to rotation of forest plantations, establishment of new orchards or the expansion/contraction of natural vegetation. Since the spatial extent of such changes was negligible the change map for the tropical study area, where changes were widespread is presented in Fig4. To corroborate the change map, Landsat images acquired around the time of SRTM and TDM passes are also shown. For an easier identification, large areas of significant positive (i.e., afforestation) or negative (i.e., deforestation) changes were highlighted on the Landsat images. Although, it was beyond the scope of this study to exhaustively test the accuracy of such changes at pixel level due to the lack of an appropriate field dataset, it is easy to recognize large patterns of forest loss or gain. The high reflectance of the near infrared (NIR) channel observed in 2000 (green tones) diminished significantly for the year 2013 for the logged areas (magenta tones). Conversely, increased NIR reflectance (i.e., greener tones) was observed for areas of afforestation or forest growth as is the case of the northernmost highlighted area or the squared shaped forest stands located in the southwestern part of the change map. Such patterns were easily identified through the positive/negative relative heights as shown in the right panel in Fig 4. The classification process was applied to about 153.000 ha, i.e., the common area of the four TDM pairs analyzed in the tropical study area. One should notice that water bodies were automatically masked out during processing since their coherence was below the 0.3 limit. The area classified as deforestation using the thresholds mentioned in Section 3.2 amounted to about 11.3% while degradation and forest growth were recorded each on about 18% of the area. About 1.6% of the area was classified as afforestation while stable areas formed the remaining 52.4% of the classified pixels. These results suggested a loss of forest for an area of about 10% over the past decade when assuming that forest growth compensates degradation. However, one should notice that areas of forest growth may have been overestimated since a positive change in the scattering center is partly attributed to the different penetration of the microwaves at X- and C-band while a negative height is less likely to be misclassified.

**Fig 5:**
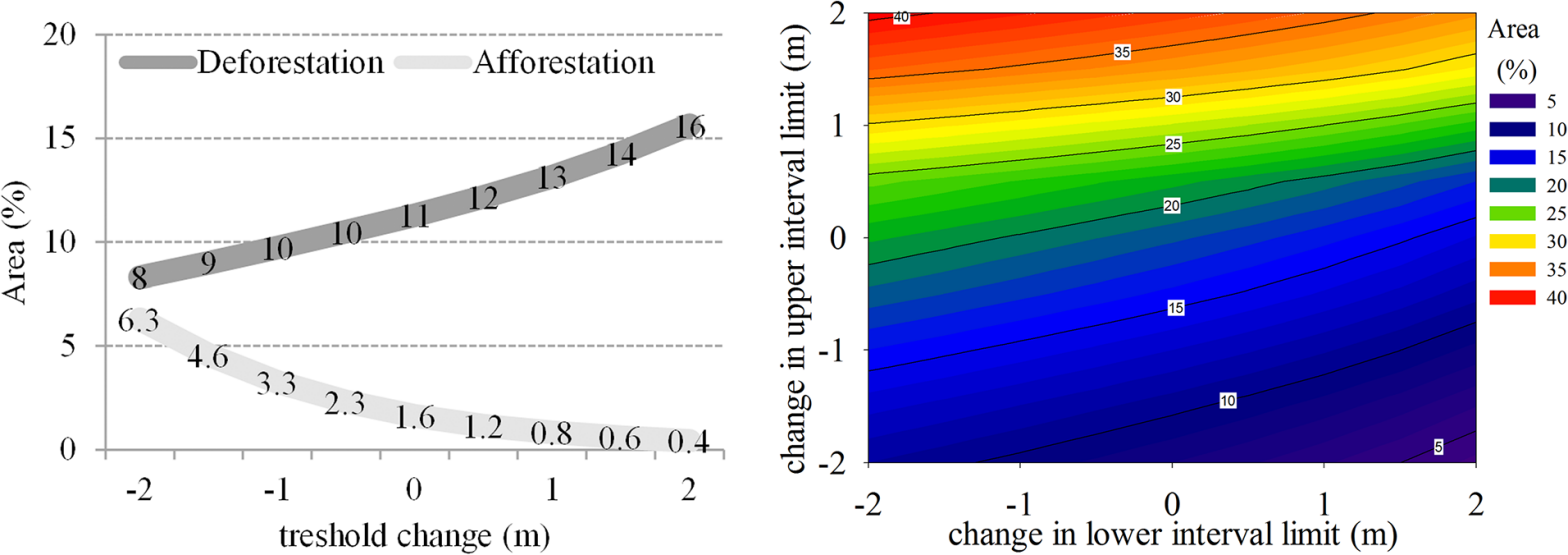
Sensitivity of deforestation and afforestation classes to changes in threshold limit (left panel). Right panel shows the sensitivity of forest degradation class to a change in upper and respectively lower limit of the interval. Zeroes represent the baseline thresholds of– 6, 8 and respectively [–6 –1] m.

The classification sensitivity to different choices of thresholds was analyzed by reclassifying the tropical study area while changing thresholds in 0.5 m steps up to ±2 m. For classes defined by intervals (i.e., degradation, stable areas and forest growth) the ± 2 m variation was applied at both ends of the interval although some of the resulting intervals would be impractical, e.g., stable areas should not present negative heights. The results showed that deforestation and afforestation classes have a limited sensitivity to reasonable changes in thresholds with the classified area varying by up to only ±5% (Fig 5, left panel). For areas affected by degradation or forest regrowth the change in area with a change in limit reached ±15% and respectively ±18% (Fig 5 right panel).

## Discussion

Observations from current space borne radar sensors operating X-, C- and L-bands contain information relative to the first few meters of the forest canopy and therefore present a partial description of the total forest structure from which carbon stocks estimates are derived. With SAR interferometry, a direct estimation of a structural parameter related to vegetation height can be obtained. Stand height is often a reliable indicator of total biomass, allowing for indirect relationships to be formed between forest height as measured by TDM and forest inventory data [12]. Nevertheless, the relative heights obtained here by comparing TDM X-band elevation and SRTM C-band elevation need calibration to account for the different location above the ground of the scattering centers at the two wavelengths. By estimating the average bias with respect to ground datasets it is possible to calibrate the relative heights and use them to quantitatively estimate changes in forest above ground biomass. Solberg et al. (2014) have recently demonstrated that DInSAR heights based on SRTM and TDM datasets may provide sufficiently accurate information to derive carbon stocks estimates. However, the exact quantification of degradation and carbon stock enhancement through forest growth requires a more careful analysis by taking into account not only the differential penetration depth of the two wavelengths but also other influencing factors such as environment or forest type and structure. Compensating for height differences due to wavelength-dependent scattering center variation might be less relevant in deforestation areas since negative heights are a strong indicator of forest loss considering that X-band scattering center is theoretically higher than C-band scattering center. In such areas, the negative height should indicate the height of the cleared forest, when accounting for the theoretical difference in the scattering centers, providing thus quantitative information on carbon stocks losses. The penetration depths (i.e., 6–10 m) observed in this study were similar with those observed for boreal forests suggesting consistent sensitivity to forest height for very different environments [12].

Overall, forest change mapping results were encouraging since change patterns were consistently identified. Moreover, the use of SAR datasets provided not only a change/no change map but also an estimate of the magnitude of such changes expressed through a decrease/increase in forest height which is extremely relevant when monitoring areas affected by degradation and carbon stock enhancements (e.g., afforestation or forest regrowth) as needed in the context of REDD+ programs. Information on height changes is relevant since an increase/decrease in stand height reflects more accurately carbon stocks gains/losses when compared to optical based maps which rely on the areal extent. Furthermore, the sensitivity to threshold selection was limited especially for deforestation and afforestation classes. An improper selection of thresholds or spatial variability in scattering center differences between the two wavelengths would change the area classified as deforestation or afforestation by only 2 to 5% for 1 to 2 m variations. Areas classified as forest degradation and growth are more prone to errors related to incorrect thresholds. However, even for such areas a change in threshold of ±1 m resulted in spatial extent differences of less than 10%. Such stable classification results are relevant particularly when carbon stocks are estimated as a function of forested area and the mean biomass per unit area. A recognized limitation of this study was the use of unique thresholds for each class which might not be appropriate since differences in scattering center may be related to local variations in forest structure or vegetation water content. Ultimately the choice of appropriate thresholds should be based on local knowledge and thoroughly validated if such change maps are to be used for management purposes or in the context of programs such as REDD.

In the context of REDD programs information on elevation differences between SRTM and TDM missions could be used in different ways: i) land cover change is identified using optical sensors while the magnitude of such changes is derived from SRTM/TDM height information, ii) SRTM/TDM data is used to simultaneously derive the spatial extent of changes as well as their magnitude as shown in this study or iii) optical and SRTM/TDM data are jointly used to derive the spatial extent of changes while their magnitude is estimated using the SRTM/TDM relative heights. Subsequently, relative heights could be used to derive changes in biomass and carbon stocks directly by estimating the increase in forest biomass for every m increase of InSAR height [35]. Such an approach, however, requires in situ data to model the relationship between the change rate in biomass and respectively InSAR height for a given forest type.

The simple methods used to produce information on the direction and magnitude of forest changes coupled with near-global availability of SRTM and TDM datasets constitutes a great incentive for using such datasets over large areas for establishing a reference trend over the past decade as well as for continued monitoring until the end of the TDM mission.

## Conclusions

This study looked at the opportunity provided by the SRTM and TDM datasets to retrieve forest height, monitor changes in forest cover and estimate the magnitude of such changes in tropical and semi-arid environments. While the variability of the retrieved InSAR relative height was largely explained by variations in ground measured height there was a large negative bias of the estimates for both study areas due to the nature of the radar datasets used. For newly established forests or plantations the relative height was considerably closer to the ground measured tree height. Combining SRTM and TDM datasets allowed not only to identify changes in forest cover but also to estimate the magnitude of such changes which is extremely relevant for monitoring forest degradation or growth. The classification sensitivity to changes in class thresholds was limited for deforestation and afforestation classes while it somewhat increased for forest degradation and forest growth classes. The concurrent use of SRTM and TDM dataset should allow for a more accurate assessment of the magnitude of forest change over the past decade providing additional support for programs such REDD and REDD+.

## Supporting Information

S1 Table Field dataset acquired for the Australian study area (September 2011). Coordinates in UTM 55 WGS84.
(PDF)

S2 Table Field dataset acquired for the Indonesian study area (February 2014). Coordinates in UTM 50N WGS84.
(PDF)
