# Supplementary material for: Detecting and Quantifying Forest Change: The Potential of Existing C- and X-Band Radar Datasets
Source: PLoS One. 2015 Jun 25;10(6):e0131079. doi: 10.1371/journal.pone.0131079 (PMC4482516; doi:10.1371/journal.pone.0131079)
Supplement: S1 Table — Coordinates in UTM 55 WGS84. (PDF) [file pone.0131079.s001.pdf]

S1 Table Field dataset acquired for the Australian study area (September 2011). Coordinates in UTM 55S WGS84

SITE ID	Longitude	Latitude	Mean Height (m)	Lorey`s height (m)	Vegetation type	Sampled area
100	413871	6153136	14.6	14.7	eucalypt plantation	15
101	413709	6152907	17.6	17.9	eucalypt plantation	15
102	413783	6153065	17.8	18.1	eucalypt plantation	15
103	405907	6152614	14.3	14.5	forest	25
104	406320	6152947	14.9	16.6	forest	25
105	406018	6153094	14.6	15.5	forest	25
106	406102	6152849	14.6	15.4	forest	25
107	406270	6153304	9.3	10.3	forest	25
108	405850	6152722	14.3	14.8	forest	25
109	405780	6152982	14.1	14.4	forest	25
201	417790	6154800	8.2	9.8	woodland	10
202	417745	6154546	8.6	8.6	woodland	10
401	409674	6146238	0.95	0	canola	50
402	414831	6159517	0.5	0	cereals	50
403	414293	6159102	0.5	0	cereals	50
404	414199	6158688	0.5	0	cereals	50
405	411270	6130376	1.15	0	canola	50
406	419122	6143019	0.48	0	grassland	50
407	415481	6157742	0.59	0	cereals	50
408	416159	6158673	0.3	0	cereals	50
601	419268	6160047	3.8	3.8	almond orchard	25
5001	418709	6160010	16.6	21	woodland	10
5002	419230	6160698	13.9	14.7	woodland	10
5003	413038	6155353	9.4	9.9	eucalypt plantation	15
5004	413665	6152895	18.9	18.7	eucalypt plantation	10
5005	414084	6152836	20.6	20.7	eucalypt plantation	10
5007	403242	6177262	4.6	4.6	olive orchard	20
