# Supplementary material for: Detecting and Quantifying Forest Change: The Potential of Existing C- and X-Band Radar Datasets
Source: PLoS One. 2015 Jun 25;10(6):e0131079. doi: 10.1371/journal.pone.0131079 (PMC4482516; doi:10.1371/journal.pone.0131079)
Supplement: S2 Table — Coordinates in UTM 50N WGS84. (PDF) [file pone.0131079.s002.pdf]

S2 Table Field dataset acquired for the Indonesian study area (February 2014). Coordinates in UTM 50N WGS84

**Site ID**	Longitude	Latitude	Mean Height (m)	Lorey`s height (m)	Vegetation type	Sampled area
**1**	230170	9752930	0.985927	0.0	shrub	10
**2**	230170	9752870	0.940968	0.0	shrub	10
**3**	228834	9752930	1.04269	0.0	shrub	10
**4**	228723	9752860	0.989697	0.0	shrub	10
**5**	229057	9752730	1.14333	0.0	shrub	10
**6**	229057	9752650	1.1112	0.0	shrub	10
**7**	224494	9752950	1.12833	0	shrub	10
**8**	224491	9754690	0.882857	0	shrub	10
**9**	224494	9752460	0.959	0.0	shrub	10
**10**	224495	9752350	1.01318	0.0	shrub	10
**11**	232840	9753260	7.26723	8.1	secondary forest	10
**12**	232840	9753300	7.50605	8.4	secondary forest	10
**13**	232951	9753310	7.57632	8.3	secondary forest	10
**14**	225859	9735610	8.02327	8.7	secondary forest	10
**15**	225748	9735620	6.45234	6.6	secondary forest	10
**16**	225637	9735680	6.83626	6.9	secondary forest	10
**17**	225525	9735790	7.41709	9.7	secondary forest	10
**18**	225747	9736090	6.88969	7.0	secondary forest	10
**19**	225636	9736170	6.76156	6.9	secondary forest	10
**20**	225635	9736320	6.66903	6.8	secondary forest	10
**21**	232394	9753630	9.08393	12.6	dense forest	10
**22**	232394	9753680	8.92485	11.3	dense forest	10
**23**	232172	9753320	8.06056	9.2	dense forest	10
**24**	231949	9753600	9.49789	13.3	dense forest	10
**25**	231838	9753360	10.5846	17.6	dense forest	10
**26**	231838	9753410	10.4065	14.3	dense forest	10
**27**	231393	9753360	9.24341	17.4	dense forest	10
**28**	231393	9753430	10.5517	16.7	dense forest	10
**29**	230837	9753290	9.0573	17.3	dense forest	10
**30**	230837	9753310	8.7061	10.7	dense forest	10
**31**	232506	9753570	9.22721	10.6	forest	10
**32**	232395	9753430	8.27857	9.8	forest	10
**33**	232061	9753440	9.24328	11.5	forest	10
**34**	232172	9753390	7.74638	8.0	forest	10
**35**	231838	9753290	10.0209	11.4	forest	10
**36**	231838	9753310	8.99445	10.3	forest	10
**37**	231393	9753270	9.23248	12.0	forest	10
**38**	231393	9753290	11.633	13.6	forest	10
**39**	230948	9753260	12.9155	15.6	forest	10
**40**	230948	9753250	8.97419	10.3	forest	10
**41**	230170	9752710	1.90618	4.9	shrub	10
**42**	230170	9752620	1.72414	4.8	shrub	10
**43**	228834	9752820	1.5676	4.5	shrub	10
**44**	228946	9752730	1.46042	5.5	shrub	10
**45**	228946	9752650	1.73074	4.5	shrub	10
**46**	228835	9752680	1.3013	3.3	shrub	10
**47**	224494	9752700	1.32714	0.0	shrub	10
**48**	224494	9752560	0.912456	0.0	shrub	10
**49**	224495	9752150	1.60524	0.0	shrub	10
**50**	224495	9751980	1.18783	0.0	shrub	10
